# A high triglyceride glucose index is associated with early renal impairment in the hypertensive patients

**DOI:** 10.3389/fendo.2022.1038758

**Published:** 2022-12-14

**Authors:** Jiankai Dong, Huijie Yang, Yaping Zhang, Lianglong Chen, Quanzhong Hu

**Affiliations:** ^1^ Department of Cardiology, Fujian Medical University Union Hospital, Fujian Cardiovascular Medical Center, Fujian Institute of Coronary Heart Disease, Fuzhou, China; ^2^ Department of Cardiology, Qinghai Provincial People’s Hospital, Xining, China

**Keywords:** hypertension, triglyceride glucose index, insulin resistance, renal impairment, β2 microglobulin, Urine microalbumin, cystatin C

## Abstract

**Objective:**

Serum β2-microglobulin (β2-MG) and serum cystatin C (CysC) are sensitive and reliable indicators of early renal impairment. Triglyceride glucose index (TyG) is an emerging vital indicator of insulin resistance and is associated with increased risk of hypertension. We aimed to analyze the relationship between TyG and early renal impairment in hypertensive patients.

**Methods:**

A retrospective analysis was performed on 881 hypertensive patients treated in Qinghai Provincial People, s Hospital from March 2018 to March 2021, their clinical data and corresponding laboratory index values were recorded, and the TyG index was calculated. According to the TyG index, the patients were divided into a low TyG (L-TyG) group (TyG ≤ 8.50, n=306), medium TyG (M-TyG) group (8.51≤TyG ≤ 8.94, n=281), and high TyG (H-TyG) group (TyG>8.95, n=294) in sequence by using tertiles. Then, according to serum β2-MG and CysC levels, they were divided into a normal renal function group (β2-MG ≤ 2.4 mg/L, n=700 and CysC ≤ 1.25mg/L, n=721) and a renal function injury group (β2-MG>2.4 mg/L, n=181, and CysC>1.25 mg/L, n=160). Multivariate linear regression analysis was used to analyze the influencing factors of serum β2-microglobulin and cystatin C. Multivariate Logistic regression was used to analyze the relationship between the TyG index and early renal impairment in hypertensive patients. The receiver operating characteristic curve (ROC) was used to determine the value of the TyG index in predicting early renal impairment in patients with hypertension.

**Result:**

As the TyG index level increased, serum β2-MG and CysC levels also gradually increased. Multivariate linear regression analysis showed that TyG index was the influencing factor of serum β2-MG (B=0.060, P=0.007) and serum CysC (B=0.096, P<0.001). For every 1 standard deviation increase in the TyG index, the serum β2-MG and CysC increased by 0.06mg/L and 0.096mg/L, respectively. When compared to the normal group, the TyG level (8.91 ± 0.65 vs 8.64 ± 0.60, P<0.001) was higher in the renal impairment group with β2-MG>2.4 mg/L. The results of multivariate logistic regression analysis revealed that for every 1 standard deviation increase in the TyG index, the risk of early renal impairment in hypertensive patients increased 1.53 times (OR=1.53, 95%CI 1.006-2.303).The ROC curves showed that the TyG index was not superior to TG in predicting early renal impairment in hypertensive patients. the AUC values were 0.623 and 0.617, respectively. Then, when CysC>1.25 mg/L was used as the renal damage group, the level of TyG was still higher than that in the normal group (8.94 ± 0.67 and 8.64 ± 0.60, P<0.001). Multivariate Logistic regression analysis showed that for every 1 standard deviation increase in the TyG index, the risk of early renal impairment in hypertensive patients increased 2.82 times (OR=2.82, 95%CI 1.863-4.262). The ROC curves showed that the TyG index was not superior to TG in predicting early renal impairment in hypertensive patients. the AUC values were 0.629 and 0.626, respectively.

**Conclusion:**

TyG index is an influential factor in serum β2-MG and CysC levels. The elevated TyG index levels are closely associated with the occurrence and development of early renal impairment in hypertensive patients, but it should be used cautiously in the prediction of early renal impairment.

## 1 Introduction

With an aging population, hypertension has become the most important risk factor for cardiovascular disease morbidity and mortality (1). In addition, hypertension is a long-term progressive chronic disease, and renal injury is its major complication. Currently, serum urea nitrogen, serum creatinine (SCr) and glomerular filtration rate (GFR) are widely used clinically to evaluate renal function. but the diagnostic specificity is low due to the high storage capacity of the kidney and the influence of drugs, age and glomerular filtration rate. Studies have shown that urine microalbumin, serum β2-microglobulin (β2-MG) and serum cystatin C (CysC) are sensitive and reliable indicators of early renal injury, and their sensitivity and specificity are higher than those of serum urea nitrogen and serum creatinine (2, 3).

Insulin resistance (IR) is common in patients with hypertension (4), and IR is strongly associated with the prevalence of chronic renal insufficiency (5). A recent study found that the triglyceride-glucose index (TyG index) derived from the log- transformation of the product of plasma triglyceride (TG) and fasting plasma glucose (FPG) was a potential IR marker (6, 7). It has been shown that an elevated TyG index is associated with the occurrence and development of atherosclerosis (8), coronary artery disease (8), hypertension (9), diabetes (10), Diabetic kidney disease (11), nonalcoholic fatty liver (12), and nephric microvascular damage (13), etc. However, there are few reports on the relationship between the TyG index and early renal injury in hypertensive patients at home and abroad.

Therefore, this study aimed to investigate the correlation between the TyG index and early renal impairment in hypertensive patients, to provide a reference basis for the early prevention, monitoring, and treatment of chronic kidney disease (CKD).

## 2 Patients & methods

### 2.1 Study population

A total of 881 inpatients with primary hypertension from March 2018 to March 2021 in Qinghai Provincial People’s Hospital were included as the subjects, including 464 males and 417 females, aged from 40 to 92 years old, with an average age of (60.06 ± 12.23) years old.

Inclusion criteria: ① Age≥40 years old, no sex limitation, and complete clinical data. ② Hypertension was defined as having two independent blood pressure measurements≥140/90mmHg or having received antihypertensive drug treatment.③ eGFR≥60 ml/min/1.73m^2^.

Exclusion criteria: secondary hypertension, coronary atherosclerotic heart disease, heart failure, diabetes; Various acute and chronic infectious diseases, primary glomerular disease, renal artery stenosis; Patients with severe liver and kidney function impairment; Thyroid disease, malignant tumor, clear familial hyperlipidemia, and lipid-lowering drugs in the past 1 month; Cognitive dysfunction, mental illness. The study protocol was approved by the Ethics Committee of Qinghai Provincial People’s Hospital. All participants signed the informed consent.

### 2.2 Data collection

Clinical data such as sex, age, body weight, smoking history, and duration of hypertension of the subjects were accurately recorded, and body mass index (BMI) was calculated as follows.


$$\text{BMI}=\text{body mass}\left(\text{kg}\right)/\text{height}{\left(\text{m}\right)}^{2}$$

All subjects fasted for 10-12 hours one day before the experiment, 4-5 mL venous blood were drawn next morning, and the serum was collected after centrifugation at 4 000 r/min. The fasting plasma glucose (FPG) was measured by the glucose oxidase method. Triglyceride (TG), urea nitrogen (BUN), serum creatinine (Scr), total cholesterol (TC), high-density lipoprotein cholesterol (HDL-C), low-density lipoprotein cholesterol (LDL-C), and serum uric acid (SUA) were measured by enzyme method. Serum β2-microglobulin (0.8-2.4mg/L) was determined by ELISA, and cystatin C (0.54-1.25mg/L) was determined by latex nephelometry. The above indexes were performed with a Beckman AU5800 automatic biochemical analyzer. The estimated glomerular filtration rate (eGFR) was calculated using the CKD-EPI formula.

### 2.3 Groups

TyG index (6)=Ln[fasting TG (mg/dL)×FBG (mg/dL)/2], where TG (1mg/dL=0.011mmol/L) and FBG (1mg/dL=0.056mmol/L). According to the TyG index, patients were divided into a low TyG (L-TyG) group (TyG ≤ 8.50, n=306), medium TyG (M-TyG) group (8.51≤TyG ≤ 8.94, n=281) and high TyG (H-TyG) group (TyG>8.95, n=294) in sequence by using tertiles. In addition, 881 hypertensive patients were divided into a normal renal function group (serum β2-MG ≤ 2.4 mg/L and CysC ≤ 1.25mg/L) and an impaired renal function group (serum β2-MG>2.4 mg/L and CysC>1.25 mg/L) according to serum β2-MG and CysC levels.

### 24 Statistical analysis

Statistical analysis was performed using SPSS 26.0 (SPSS Inc.). The single-sample k-s test (two-sided test) was used to test whether the measurement data conformed to a normal distribution. Measurement data of normal distribution are expressed as the mean ± standard deviation. Student’s t-test was used for comparisons between two groups, a one-way analysis of variance was used for comparisons among three groups, and the LSD test was used for pairwise comparisons between groups. The measurement data with a nonnormal distribution are expressed as M(Q25, Q75). Means of 2 groups were compared using the Mann-Whitney U test. Multiple-group comparisons were made by Kruskal-Wallis tests. Enumeration data were compared using the X^2^ test. Multifactor linear stepwise regression was used to analyze the influencing factors of serum β2- microglobulin and cystatin C. Logistic regression analysis was used to analyze the influencing factors of early renal impairment in patients with hypertension. The receiver operating characteristic curve (ROC) was used to determine the value of the TyG index in predicting early renal impairment in patients with hypertension. P<0.05 was considered statistically significant.

## 3 Results

### 3.1 Comparison of general data and clinical indicators among three groups of TyG index

A total of 881 patients (mean age 60.06 ± 12.23 years, 464 men) with hypertension were included in the study. The baseline clinical characteristics and laboratory measurements of patients within the groups are presented in **Table 1**. Participants were grouped into tertiles according to their TyG level. There were no significant differences among the three groups in sex, smoking history, BMI, hypertension course, HS-CRP, mALB, BUN and eGFR levels (P>0.05). The levels of TC, HDL-C, LDL-C, Alb, TG, FBG, β2-MG, CysC and SUA were compared and the differences were statistically different (P<0.05, as shown in **Table 1**). Besides, the levels of β2-MG (1.96 ± 0.82) and CysC (1.04 ± 0.27) in the M-TyG group were significantly higher than that in the L-TyG group (1.87 ± 0.65 and 1.03 ± 0.44). The levels in the H-TyG group (2.29 ± 1.04 and 1.18 ± 0.44) were significantly higher than that in the M-TyG group (1.96 ± 0.82 and 1.04 ± 0.27).

**Table 1:** Comparison of general data and clinical indicators of the L-TyG group, M-TyG group, and H-TyG.

	L-TyG group (n=306)	M-TyG group (n=281)	H-TyG group (n=294)	P value
Male [n,(%)]	175 (57.2%)	135 (48.0%)	155 (52.7%)	0.086
Smoking history [, (%)]	64 (20.9%)	54 (19.2%)	80 (27.2)	0.052
Age (y)	62.00 ± 12.92	60.53 ± 11.85	57.61 ± 11.47^ab^	<0.001
BMI (kg/m2)	25.09 ± 4.11	25.59 ± 3.79	25.64 ± 3.62	0.153
hypertension course (months)	60.00 (36.00-120.00)	60.00 (24.00-120.00)	54.00 (24.00-120.00)	0.103
TC (mmol/l)	3.97 ± 0.89	4.32 ± 0.83^a^	4.38 ± 2.22^ab^	<0.001
HDL-C (mmol/L)	1.11 ± 0.29	1.05 ± 0.22^a^	0.97 ± 0.20^ab^	<0.001
LDL-C (mmol/L)	2.40 ± 0.74	2.68 ± 0.70^a^	2.72 ± 0.77^a^	<0.001
Alb (mg/L)	39.05 ± 4.24	40.22 ± 3.86^a^	40.88 ± 3.93^a^	<0.001
Scr (μmol/L)	68.48 ± 14.17	69.57 ± 13.64	71.59 ± 14.93^a^	0.026
eGFR[ml.min-1.(1.73m2)-1]	94.96 ± 16.13	92.68 ± 16.64	92.93 ± 17.76	0.179
HS-CRP (mg/dl)	0.13 (0.06-0.33)	0.14 (0.06-0.29)	0.15 (0.08-0.30)	0.465
BUN (mmol/L)	5.75 ± 1.67	5.49 ± 1.51	5.57 ± 1.42	0.138
SUA (μmol/L)	347.54 ± 109.99	358.45 ± 96.54	385.25 ± 104.74^ab^	<0.001
TG (mmol/L)	0.92 ± 0.29	1.63 ± 0.32^a^	2.99 ± 0.84^ab^	<0.001
FBG (mmol/L)	4.50 ± 0.61	4.78 ± 0.56^a^	5.20 ± 0.53^ab^	<0.001
mALB (mg/L)	1.00 (0.59-2.45)	1.06 (0.61-2.16)	1.11 (0.59-2.36)	0.957
β2-MG (mg/L)	1.87 ± 0.65	1.96 ± 0.82	2.29 ± 1.04^ab^	<0.001
CysC (mg/L)	1.03 ± 0.44	1.04 ± 0.27	1.18 ± 0.44^ab^	<0.001

Data are expressed as the mean ± SD, median (25th–75th percentiles), or number (percentage). BMI, body mass index; Fib, fibrinogen;TC, cholesterol; HDL-C, high-density lipoprotein cholesterol level; LDL-C, low-density lipoprotein cholesterol level; Alb,albumin; Scr, serum creatinine; eGFR, estimated glomerular filtration rate; HS-CRP, high-sensitivity C-reactive protein;BUN, Blood Urea Nitrogen; SUA, Serum uric acid; TG, triglyceride;FBG, fasting blood-glucose; mALB, urine microalbumin; β2-MG, serum β2 microglobulin; CysC, Serum cystatin C.

a p < 0.05 compared with the L-TyG group; p<0.01 compared with the M-TyG group.

### 3.2 Multivariate linear regression analysis

The relationship between the TyG index and early renal injury indicators was analyzed by Multivariate linear regression with serum β2-MG and serum cystatin C as dependent variables and age, BMI, TC, hypertension course, LDL-C, Alb, HS-CRP, HDL-C, eGFR, SUA, TyG index, BUN, mALB and Sex as independent variables. The results showed that the TyG index level was an influencing factor of serum β2-MG (B=0.060, 95%CI 0.017-0.103, P=0.007) and CysC (B=0.096, 95%CI 0.055-0.137, P<0.001). For every 1 standard deviation increase in the TyG index, the serum β2-MG and CysC increased by 0.06mg/L and 0.096mg/L, respectively (**Table 2**).

**Table 2:** Multivariate linear regression analysis.

Dependent variable	Independent variable	B	SE	Beta	t	P value	95% CI
β2-MG	Age	0.004	0.001	0.113	2.811	0.005	(0.001, 0.006)
	BMI	0.005	0.003	0.047	1.519	0.129	(-0.001, 0.011)
	hypertension course	0.001	0.002	0.01	0.307	0.759	(-0.003, 0.004)
	TC	0.01	0.006	0.057	1.794	0.073	(-0.001, 0.022)
	LDL-C	-0.007	0.018	-0.014	-0.414	0.679	(-0.042, 0.027)
	mALB	0	0.001	0.011	0.348	0.728	(-0.002, 0.003)
	eGFR	-0.006	0.001	-0.262	-6.508	<0.001	(-0.008, -0.004)
	Alb	-0.006	0.003	-0.058	-1.73	0.084	(-0.012, 0.001)
	HS-CRP	0.083	0.017	0.152	4.91	<0.001	(0.05, 0.117)
	SUA	0.001	0	0.136	3.671	<0.001	(0, 0.001)
	BUN	0.007	0.009	0.026	0.793	0.428	(-0.01, 0.024)
	HDL-C	-0.132	0.055	-0.082	-2.399	0.017	(-0.24, -0.024)
	TyG index	0.060	0.022	0.092	2.714	0.007	(0.017, 0.103)
	Sex (Male)	0.071	0.029	0.088	2.408	0.016	(0.013, 0.129)
CysC	Age	0.003	0.001	0.095	2.357	0.019	(0, 0.005)
	BMI	-0.003	0.003	-0.034	-1.102	0.271	(-0.009, 0.003)
	hypertension course	0.001	0.002	0.018	0.542	0.588	(-0.003, 0.004)
	TC	-0.008	0.006	-0.046	-1.422	0.155	(-0.019, 0.003)
	LDL-C	0.012	0.017	0.023	0.71	0.478	(-0.021, 0.045)
	mALB	0.003	0.001	0.075	2.461	0.014	(0.001, 0.005)
	eGFR	-0.006	0.001	-0.256	-6.342	<0.001	(-0.008, -0.004)
	Alb	-0.008	0.003	-0.087	-2.591	0.010	(-0.014, -0.002)
	HS-CRP	0.046	0.016	0.088	2.836	0.005	(0.014, 0.078)
	SUA	0	0	0.131	3.529	<0.001	(0, 0.001)
	BUN	0.019	0.008	0.075	2.252	0.025	(0.002, 0.035)
	HDL-C	0.058	0.053	0.038	1.11	0.267	(-0.045, 0.162)
	TyG index	0.096	0.021	0.155	4.545	<0.001	(0.055, 0.137)
	Sex(Male)	-0.054	0.028	-0.07	-1.913	0.056	(-0.109, 0.001)

Abbreviations as given in **Table 1**.

### 3.3 Comparison of general data and clinical index between two groups on β2-MG level

Patients were divided into a normal renal function group (β2-MG ≤ 2.4 mg/L) and an impaired renal function group (β2-MG>2.4 mg/L) according to β2-MG level. The age of patients in the renal impairment group (66.54 ± 12.19) was significantly higher than that in the normal renal function group (58.40 ± 11.69, P<0.001), and there was no significant difference in the levels of sex, smoking history, BMI, TC, LDL-C or mALB between the two groups (P>0.05). The levels of hypertension course, Scr, HS-CRP, BUN, SUA, TG and TyG index in the renal function injury group were significantly higher than those in the normal renal function group. HDL-C, Alb, and eGFR levels were significantly lower than those in the normal renal function group, and the differences were statistically significant (P<0.05, see **Table 3**).

**Table 3:** Comparison of general data and clinical index between two groups on β2-MG level.

	Normal renal function group (n=700)	Impaired renal function group (n=181)	P value
Male [n,(%)]	380 (54.3%)	85 (47.0%)	0.079
Smoking history [n, (%)]	163 (23.3%)	35 (19.3%)	0.257
Age (y)	58.40 ± 11.69	66.54 ± 12.19	<0.001
BMI(kg/m2)	25.31 ± 3.71	25.93 ± 4.36	0.084
hypertension course (months)	48.00 (24.00-120.00)	84.00 (36.00-132.00)	0.001
TC (mmol/l)	4.30 ± 0.89	4.72 ± 4.57	0.219
HDL-C (mmol/L)	1.06 ± 0.24	0.99 ± 0.29	0.001
LDL-C (mmol/L)	2.59 ± 0.73	2.62 ± 0.83	0.640
Alb (mg/L)	40.40 ± 3.96	38.63 ± 4.30	<0.001
Scr (μmol/L)	68.32 ± 13.63	75.87 ± 15.31	<0.001
eGFR [ml.min-1.(1.73m2)-1]	96.91 ± 15.58	80.60 ± 15.35	<0.001
HS-CRP (mg/dl)	0.12 (0.06-0.26)	0.24 (0.11-0.56)	<0.001
BUN (mmol/L)	5.48 ± 1.46	6.12 ± 1.77	<0.001
SUA (μmol/L)	353.31 ± 96.08	403.43 ± 127.48	<0.001
TG (mmol/L)	1.75 ± 0.96	2.22 ± 1.18	<0.001
FBG (mmol/L)	4.79 ± 0.65	4.97 ± 0.58	0.001
TyG index	8.64 ± 0.60	8.91 ± 0.65	<0.001
mALB (mg/dl)	1.01 (0.60-2.07)	1.19 (0.63-3.22)	0.051

Data are expressed as the mean ± SD, median (25th–75th percentiles), or number (percentage). TyG index, Triglyceride glucose index.

### 3.4 Comparison of general data and clinical index between two groups on the level of CysC

Patients were divided into a normal renal function group (CysC ≤ 1.25 mg/L) and an impaired renal function group (CysC>1.25 mg/L) according to CysC level. There was no significant difference in the levels of sex, BMI, TC, HDL-C, LDL-C or mALB levels between the two groups (P>0.05). The levels of age, hypertension course, Scr, HS-CRP, BUN, SUA, TG, and TyG index in the renal impairment group were significantly higher than those in the normal renal function group. The smoking history, Alb, and eGFR levels were significantly lower than those in the normal renal function group, and the differences were statistically significant (P<0.05, see **Table 4**).

**Table 4:** Comparison of general data and clinical index between two groups on the CysC level.

	Normal renal function group (n=721)	Impaired renal function group (n=160)	P value
Male [n,(%)]	369 (51.2%)	95 (59.4%)	0.063
Smoking history [n, (%)]	150 (20.8%)	47 (29.4%)	0.019
Age (y)	58.55 ± 11.74	66.94 ± 12.12	<0.001
BMI (kg/m^2^)	25.43 ± 3.72	25.47 ± 4.45	0.927
hypertension course(months)	54.00 (24.00-120.00)	72.00 (36.00-153.00)	0.001
TC (mmol/l)	4.39 ± 2.40	4.39 ± 1.07	0.996
HDL-C (mmol/L)	1.06 ± 0.25	1.02 ± 0.26	0.122
LDL-C (mmol/L)	2.59 ± 0.73	2.65 ± 0.83	0.395
Alb (mg/L)	40.38 ± 3.95	38.50 ± 4.38	<0.001
Scr (μmol/L)	67.78 ± 13.83	79.25 ± 12.68	<0.001
eGFR [ml.min^-1^.(1.73m^2^)^-1^]	96.61 ± 15.85	79.72 ± 14.21	<0.001
HS-CRP (mg/dl)	0.13 (0.06-0.28)	0.22 (0.10-0.52)	<0.001
BUN (mmol/L)	5.44 ± 1.44	6.39 ± 1.75	<0.001
SUA (μmol/L)	352.42 ± 97.91	414.28 ± 121.40	<0.001
TG (mmol/L)	1.74 ± 0.94	2.32 ± 1.26	<0.001
FBG (mmol/L)	4.80 ± 0.63	4.95 ± 0.65	0.008
TyG index	8.64 ± 0.60	8.94 ± 0.67	<0.001
mALB (mg/L)	1.03 (0.60-2.10)	1.18 (0.63-3.52)	0.107

Data are expressed as mean ± SD, median (25th–75th percentiles), or number (percentage). TyG index, Triglyceride glucose index.

### 3.5 Multi-factor logistic regression analysis of influencing factors of early renal impairment in patients with hypertension

Taking the occurrence of renal impairment (β2-MG>2.4 mg/L) as the dependent variable, and taking age, BMI, total cholesterol, hypertension course, LDL-C, Alb, HS-CRP, HDL-C, eGFR, SUA, TyG index, BUN, mALB, and Sex as the independent variables, a multivariate Logistic regression analysis was performed. The results showed that age (OR=1.03, 95%CI 1.011-1.055, P=0.003), TyG index (OR=1.53, 95%CI 1.008-2.319, P=0.046), HS-CRP (OR=1.81, 95%CI 1.349-2.415, P<0.001), SUA (OR=1.01, 95%CI 1.002-1.006, P=0.001), HDL-C (OR=0.29, 95%CI 1.349-2.415, P=0.014), eGFR (OR=0.95, 95%CI 0.932-0.964, P<0.001) and Male (OR=0.62, 95%CI 0.385-0.989, P=0.045), these were the risk factors for early renal impairment in patients with hypertension (P<0.05, see **Table 5**).

**Table 5:** Multivariate Logistic regression analysis of early renal impairment (β2-MG>2.4mg/L) in hypertensive patients.

	β	SE	Wald X^2^ value	P value	OR value	95%CI
Age	0.032	0.011	8.689	0.003	1.03	(1.011, 1.055)
BMI	0.046	0.026	3.163	0.075	1.05	(0.995, 1.101)
hypertension course	0	0.014	0.001	0.973	1	(0.974, 1.027)
TyG index	0.425	0.212	3.994	0.046	1.53	(1.008, 2.319)
TC	0.486	0.251	3.747	0.053	1.63	(0.994, 2.659)
LDL-C	-0.479	0.284	2.837	0.092	0.62	(0.355, 1.082)
Alb	-0.051	0.030	2.866	0.090	0.95	(0.896, 1.008)
HS-CRP	0.590	0.149	15.800	<0.001	1.81	(1.349, 2.415)
HDL-C	-1.243	0.506	6.037	0.014	0.29	(0.107, 0.778)
eGFR	-0.054	0.008	39.900	<0.001	0.95	(0.932, 0.964)
SUA	0.004	0.001	11.153	0.001	1.01	(1.002, 1.006)
BUN	0.015	0.070	0.045	0.832	1.02	(0.885, 1.163)
mALB	0.001	0.008	0.007	0.932	1.00	(0.986, 1.105)
Sex (Male)	-0.482	0.241	4.019	0.045	0.62	(0.385, 0.989)

Abbreviations as given in **Table 1**.

Then, renal impairment (CysC>1.25mg/L) was used as the dependent variable. A multivariate Logistic regression analysis was performed. The results showed that age (OR=1.04, 95%CI 1.015-1.062, P=0.001), TyG index (OR=2.98, 95%CI 1.954-4.535, P<0.001), HS-CRP (OR=1.40, 95%CI 1.095-1.792, P=0.007), SUA (OR=1.01, 95%CI 1.001-1.006, P=0.002), Alb (OR=0.93, 95%CI 0.871-0.985, P=0.015), eGFR (OR=0.95, 95%CI 0.938-0.970, P<0.001) and Male (OR=1.88, 95%CI 1.114-3.038, P=0.013), these were the risk factors for early renal impairment in patients with hypertension (P<0.05, see **Table 6**).

**Table 6:** Multivariate Logistic regression analysis of early renal impairment (CysC>1.25mg/L) in hypertensive patients.

	β	SE	Wald X^2^ value	P value	OR value	95%CI
Age	0.038	0.012	10.387	0.001	1.04	(1.015, 1.062)
BMI	-0.19	0.028	0.462	0.497	0.99	(0.929, 1.036)
hypertension course	0.004	0.014	0.080	0.778	1.01	(0.977, 1.032)
TyG index	1.091	0.215	25.774	<0.001	2.98	(1.954, 4.535)
TC	-0.061	0.094	0.418	0.518	0.94	(0.782, 1.132)
LDL-C	0.080	0.174	0.214	0.644	1.09	(0.771, 1.524)
Alb	-0.076	0.032	5.884	0.015	0.93	(0.871, 0.985)
HS-CRP	0.337	0.126	7.197	0.007	1.40	(1.095, 1.792)
HDL-C	0.683	0.457	2.235	0.135	1.98	(0.809, 4.846)
eGFR	-0.048	0.009	31.172	<0.001	0.95	(0.938, 0.970)
SUA	0.004	0.001	9.987	0.002	1.01	(1.001, 1.006)
BUN	0.127	0.071	3.160	0.075	1.14	(0.987, 1.305)
mALB	0.013	0.009	2.006	0.157	1.013	(0.995, 1.032)
Sex (Male)	0.630	0.253	6.217	0.013	1.878	(1.144, 3.083)

Abbreviations as given in **Table 1**.

### 3.6 ROC curve of TyG index in predicting early renal impairment in hypertensive patients

**Figure 1:**
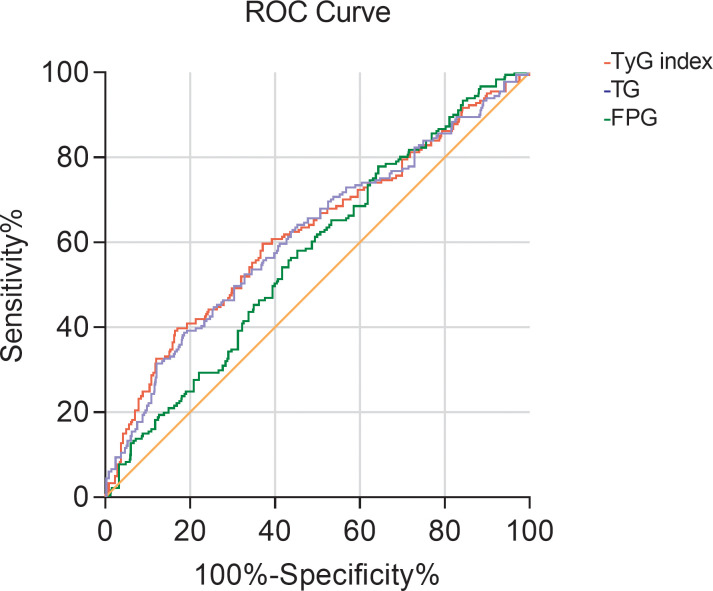
The ROC curve of TG, FBG, and TyG index for predicting early renal impairment (β2-MG>2.4mg/L) in patients with hypertension.

The ROC curve of the TyG index predicting early renal impairment in hypertensive patients was further drawn. The renal impairment was defined as β2-MG > 2.4 mg/L. The results showed that the area under the ROC curve of the TyG index in predicting early renal impairment in hypertensive patients was 0.623 (P<0.001; 95%CI0.574-0.671), when the TyG index is 9.205, the Jouden index is 0.225, the sensitivity is 39.2%, and the specificity is 83.3%. The AUC of the FPG and TG levels for predicting early renal impairment occurs in hypertensive patients was 0.573 (95%CI 0.528-0.618, P=0.002) and 0.617 (95%CI 0.569-0.665, P<0.001), respectively (**Figure 1**).

**Figure 2:**
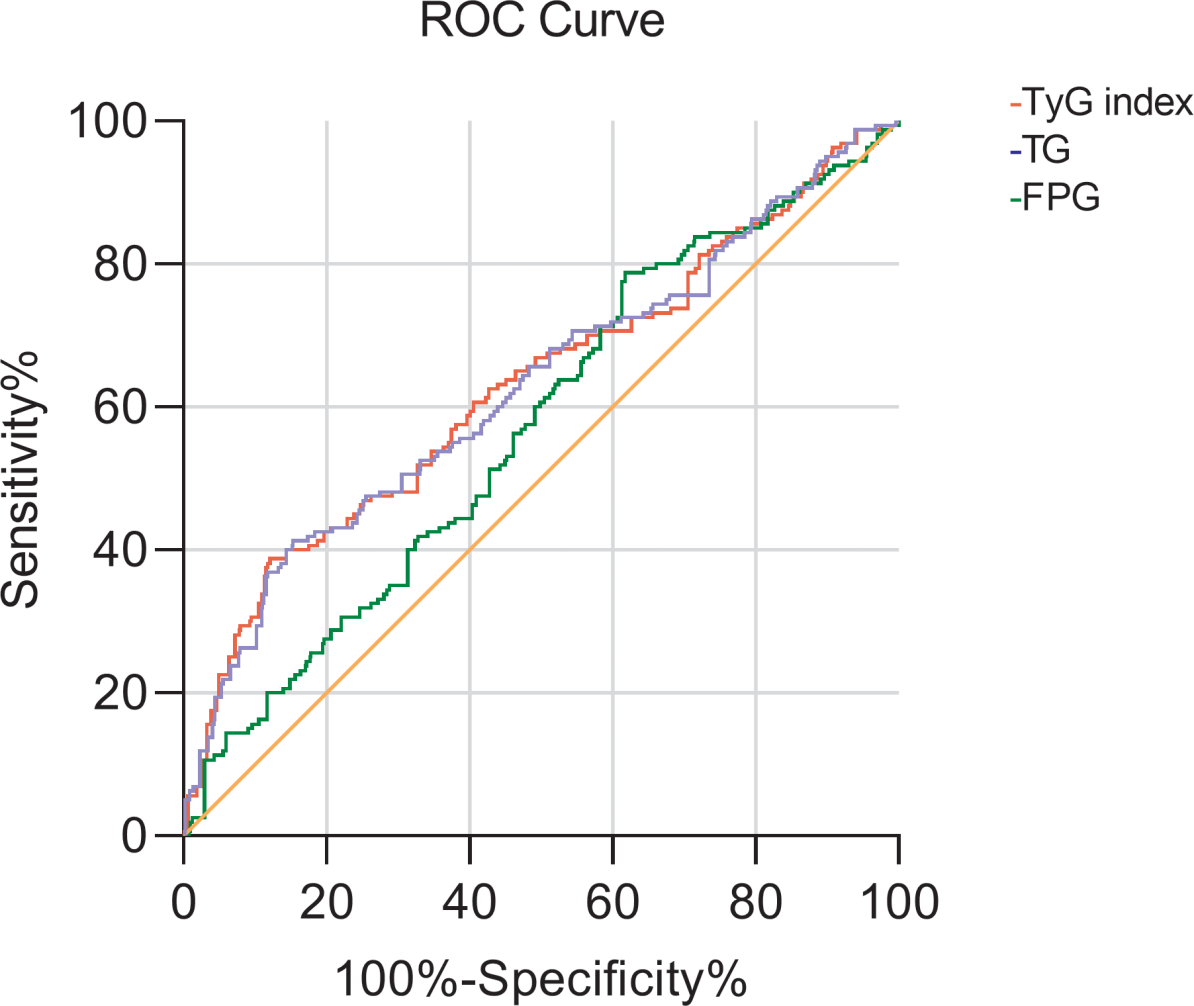
The ROC curve of TG, FBG, and TyG index for early renal impairment (CysC>1.25mg/L) in patients with hypertension.

Then with CysC>1.25 mg/L as the occurrence of renal impairment, the ROC curve of the TyG index predicting early renal impairment in hypertensive patients was also drawn, and the area under the curve was 0.629 (P<0.001; 95%CI 0.577-0.681), when the TyG index is 9.315, the maximum Jouden index is 0.266, the sensitivity is 38.8%, and the specificity is 87.8%. The AUC of the FPG and TG levels for predicting early renal impairment occurs in hypertensive patients was 0.570 (95%CI 0.522-0.619, P=0.005) and 0.626 (95%CI 0.574-0.678, P<0.001), respectively (**Figure 2**).

## 4 Discussion

This study investigated the relationship between the TyG index and early renal impairment in hypertensive patients. The results of Multivariate linear regression analysis showed that the level of TyG index was an influencing factor of serum β2-MG and CysC. Logistic regression analysis showed that the relative risk of developing renal impairment was higher in patients with elevated TyG index, suggesting that the TyG index was significantly associated with early renal impairment in hypertensive patients.

CKD is associated with increased mortality, and one of the factors contributing to increased mortality is IR. IR is present in patients with early CKD and progressively worsens as the disease progresses to end-stage renal disease. IR may participate in the occurrence and development of CKD through such mechanisms as a hyperinsulinemic reaction (14), induction of oxidative stress (15), activation of a sympathetic renin-angiotensin-aldosterone system (16), activation of inflammatory factors (17), and inhibition of insulin signaling pathway (18). Cheng HT al (19). have found that IR plays an important role in the occurrence and development of renal lesions in chronic renal diseases, which is associated with a rapid decline in the prevalence and renal function of CKD.

Microalbuminuria is an early manifestation of hypertensive renal injury and can be used as an early indication of renal injury (20). Urinary microalbumin cannot pass the glomerular filtration membrane under physiological conditions, and its content is extremely low. When the glomerulus is damaged, the protein filtration barrier will be damaged, with increased permeability and excretion of microalbumin in urine. Hence, microalbuminuria is one of the sensitive indicators of early glomerular injury (21). However, the random urine test has mixed influencing factors, such as urine concentration and exercise, which can affect its test results, resulting in poor applicability of clinical diagnosis. In addition, BUN and SCr are widely used clinically to evaluate renal function. However, due to the strong renal storage capacity, as well as the influence of drugs, age, glomerular filtration rate and other factors, significant abnormalities only appear in severe renal impairment, and its diagnostic specificity is low in the early stage of renal impairment. Therefore, in this study, the correlation between the TyG index and markers of renal disease such as mAlb, eGRF and BUN was small. CysC is a cysteine protease inhibitor, produced by nucleated cells. This molecule is widely present in tissues throughout the body and is produced at a constant rate (22). The kidney is the only organ of CysC clearance and the glomerular filtration rate determines its concentration in the serum. Therefore, CysC is a sensitive indicator reflecting the glomerular filtration rate (23). The previous study has found that the sensitivity and specificity of CysC in the diagnosis of kidney injury were superior to those of creatinine and urea nitrogen, and it had higher sensitivity and stronger specificity, which gradually increased with the aggravation of the disease (24). Meanwhile, Khan et al. (25) studied 300 patients with CKD aged ≥65 and found that CysC was not related to body mass index and age of patients, and CysC had better performance than serum creatinine in assessing the renal function of patients, which is consistent with the results of this study. β2-MG is a small molecular protein with low content in the body, but the concentration remains constant. β2-MG is excreted through the glomerular filtration membrane, and decreased glomerular filtration rate can lead to increased β2-MG level, suggesting impairment of renal function. Studies have confirmed that the abnormal rate of β2-MG reaches 42.07% when the serum creatinine is increased by 0.7%, which is a sensitive indicator of early lesions in hypertensive kidney injury (26).

IR is an underlying condition for hypertension onset (27). The current gold standard for IR measurement is the normal blood glucose clamp test. However, due to the time-consuming, expensive, and complex nature of glucose clamp testing, it is difficult to apply in large-population research and clinical settings. Guerrero-Romero et al. (7) investigated a TyG index derived from fasting triglyceride and glucose concentrations as an IR index and validated it against the normoglycemic clamp test in a Mexican population, showing its superiority over HOMA-IR. Studies have confirmed that the TyG index predicts the development of hypertension in Chinese populations (28) and may reflect cardiac remodeling, dysfunction, and atherosclerosis (29). KHAN et al. (30) found a high positive correlation between the TyG index and urine albumin creatine ratio in a cross-sectional study that included 227 patients with metabolic syndrome. Fritz et al. (31)found that the TyG index appeared to be associated with ESKD risk and mediates nearly half of the total association between BMI and ESKD in the general population. This study was the first to explore the relationship between the TyG index and early renal impairment in hypertensive patients. Because of the special geographical location of our hospital in the northwest plateau, we have a medical center for hypertension, so our study mainly included patients whose blood pressure was unstable at an early stage, and who did not have heart disease, tumors, etc. This is the advantage of our study, which can better assess the relationship between the TYG index and early renal function in hypertensive patients. The results showed that markers of early renal impairment such as serum β2-MG and CysC in the H-TyG index group were significantly higher than those in the M-TyG group and L-TyG group, and the level in the M-TyG group was also higher than that in the L-TyG group. However, the results of this study showed that the TyG index was not related to urinary microalbumin, which could be affected by several confounding factors. In addition, β2-MG>2.4 mg/L and CysC>1.25 mg/L in serum were considered as the early stage of renal impairment. All the results have shown that the TyG index level in the renal impairment group was significantly higher than that in the normal renal function group. The results of the ROC curve analysis showed that the TyG index had some value in predicting early renal impairment in hypertensive patients, but was not superior to TG alone. TyG index is the product index of TG and FBG, which is respectively an index of blood glucose and lipid metabolism, reflecting well the fat toxicity and sugar toxicity playing very important roles in Diabetic Kidney Disease.

## Study limitations

This study has certain limitations. First, results were obtained from a retrospective study, and selection bias was inevitable. Second, we only measured fasting triglycerides and fasting plasma glucose levels at admission; these parameters were not measured continuously. Third, we can only prove the correlation between the TyG index and early renal impairment in patients with hypertension, but whether they have a causal relationship requires further prospective studies.

## Conclusion

TyG index is an influential factor in serum β2-MG and CysC levels. The elevated TyG index levels are closely associated with the occurrence and development of early renal impairment in hypertensive patients, but it should be used cautiously in the prediction of early renal impairment. Therefore, for hypertensive patients, to protect target organ function, the treatment of IR should be paid attention to delay the occurrence of renal damage while strictly controlling blood pressure to reach the standard.

## Data availability statement

The original contributions presented in the study are included in the article/supplementary material. Further inquiries can be directed to the corresponding authors.

## Ethics statement

The studies involving human participants were reviewed and approved by Qinghai Provincial People’s Hospital. The patients/participants provided their written informed consent to participate in this study.

## Author contributions

JD and HY: Data collection, analyses and interpretation, and writing of the final manuscript. JD, HY, YZ, LC, and QH: Study design, Data interpretation, and revising the manuscript. All authors contributed to the article and approved the submitted version.
